# Long-term pulmonary repair in rat lungs after sublobar resection: electrocautery versus stapler methods

**DOI:** 10.1007/s11748-024-02098-8

**Published:** 2024-10-28

**Authors:** Shunichiro Matsuoka, Daisuke Hara, Daisuke Nakamura, Hirotaka Kumeda, Kentaro Miura, Mai Iwaya, Takashi Eguchi, Kazutoshi Hamanaka, Takeshi Uehara, Kimihiro Shimizu

**Affiliations:** 1 Division of General Thoracic Surgery, Department of Surgery, Shinshu University School of Medicine, 3-1-1 Asahi, Matsumoto, 390-8621 Japan; 2 Department of Laboratory Medicine, Shinshu University School of Medicine, 3-1-1 Asahi, Matsumoto, 390-8621 Japan

**Keywords:** Sublobar lung resection, Electrocautery, Stapler, Lung self-repair

## Abstract

**Objective:**

We investigated and compared the long-term (6-month) histologic changes in a rat model of sublobar resection created using electrocautery or stapler techniques.

**Methods:**

Nine-week-old male rats were anesthetized and intubated; thoracotomy with sublobar resection was performed in the right middle lobe using electrocautery or stapler techniques. Histological examination was performed at 2, 4, 8, 12, and 24 weeks post-surgery to assess long-term effects on lung tissue repair and morphologic changes. Lung expansion and alveolar epithelial cell proliferation were evaluated by measuring the mean linear intercept and counting the number of alveolar type I and II cells.

**Results:**

The electrocautery group showed signs of lung self-repair at the resected area over time, with inflammatory cell infiltration followed by growth of vessels and bronchioles. Mesothelial cells covered the resected area by 2 weeks; elastic fibers gradually connected from both sides by 24 weeks. Lung expansion, measured by mean linear intercept, was initially small below the electrocautery resection area at 2 weeks but recovered from 4 to 24 weeks. The stapler group showed persistently small mean linear intercept over time. In the electrocautery group, the number of alveolar type II cells was higher just below the resection than in other areas from 2 to 24 weeks, followed by alveolar type I cells (4 to 24 weeks). The stapler group showed a transient alveolar type II cell increase at 2 weeks.

**Conclusions:**

Compared to the stapler technique, electrocautery may provide advantages for postoperative lung repair by promoting lung expansion and alveolar epithelial cell proliferation.

**Supplementary Information:**

The online version contains supplementary material available at 10.1007/s11748-024-02098-8.

## Introduction

Owing to the advances in imaging technology, early-stage non-small-cell lung cancer (NSCLC) can now be more readily detected. Several retrospective studies have reported the efficacy of lung-preserving resection methods, such as wedge resection and segmentectomy, for selected patients [1–3]. Notably, a recent large-scale prospective trial (JCOG0802/WJOG4607L) found that patients with early-stage NSCLC who underwent segmentectomy had better overall survival than those who underwent lobectomy [4]. Another significant prospective trial (CALGB140503) that compared sublobar resection, including wedge resection and segmentectomy, further supported this trend [5]. Based on these results, the use of sublobar resection is anticipated to increase worldwide.

During the JCOG0802/WJOG4607L trial, postoperative respiratory function was used as the secondary endpoint. One year after surgery, the respiratory function of the segmentectomy group was significantly better than that of the lobectomy group. However, it is important to highlight that the observed difference in respiratory function between the two groups was relatively small, measuring only 3.5%. This difference did not meet the predefined threshold of clinical significance, which was set to 10%. Despite ongoing discussions regarding this finding, uncertainty regarding its clinical implications remains.

Segmentectomy presents various surgical challenges. One critical issue is determining whether the electrocautery or stapler technique is better for dividing the intersegmental plane. The electrocautery technique is the traditional approach to dividing the intersegmental veins [6–8]. However, previous studies have reported that staplers can reduce postoperative complications, such as air leaks, compared to electrocautery [9, 10]. Conversely, a large study of 396 patients reported that the use of electrocautery did not affect the development of postoperative complications in selected patients [11]. The use of a stapler has been associated with postoperative peri-staple atelectasis and hematomas [12, 13], which can lead to inadequate postoperative lung function. Electrocautery is believed to offer the advantages of precise division of the intersegmental plane and preservation of the shape of the residual segments. However, no studies have examined the morphologic changes in the preserved lung lobes after the use of both methods. During this study, we aimed to investigate the long-term histologic features of preserved lung lobes following the application of the electrocautery and stapler techniques using a rat sublobar resection model.

## Materials and methods

### Rat models of sublobar resection using the electrocautery and stapler techniques

**Fig. 1:**
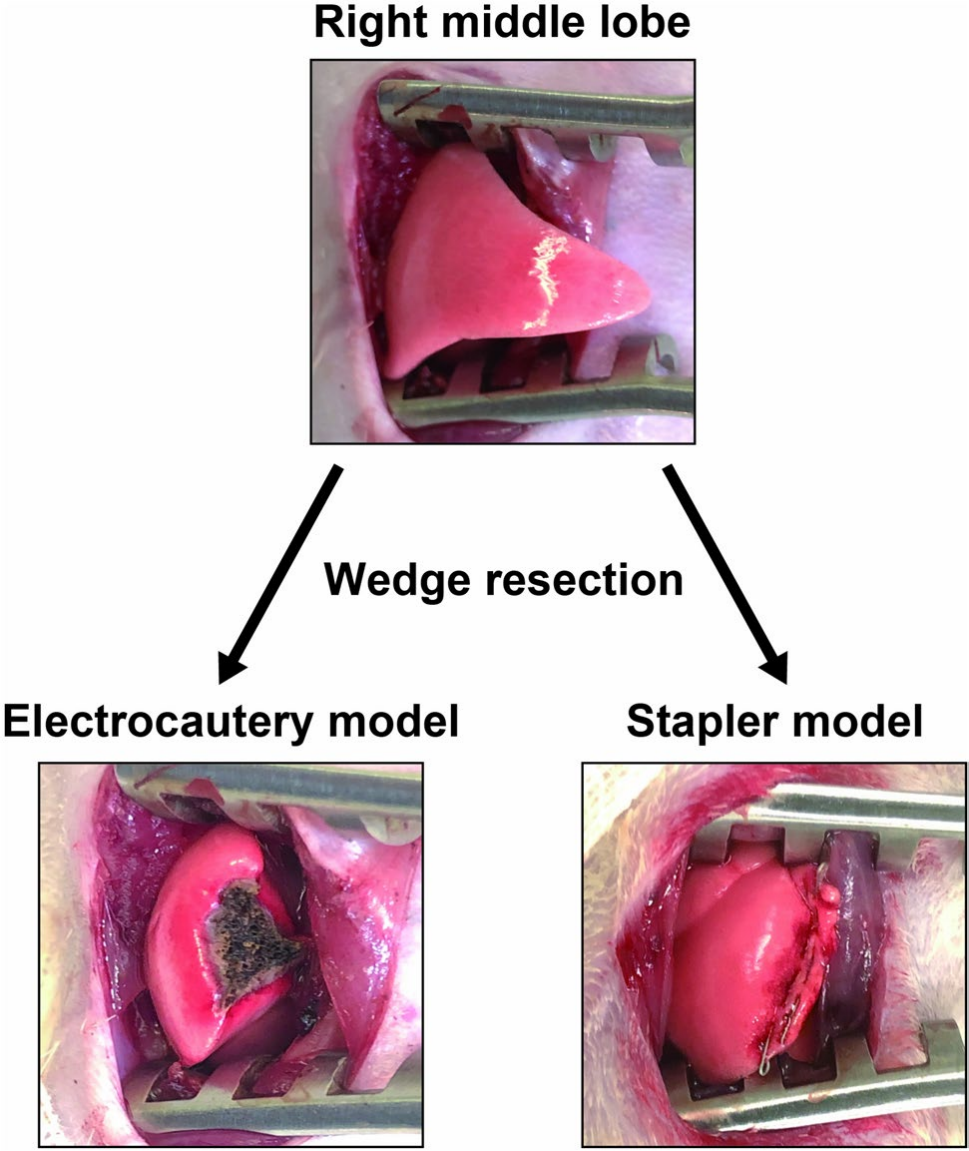
Rat sublobar resection model created using electrocautery and stapler techniques, showing the right middle lobes of the rat before and after sublobar resection

Nine-week-old male Sprague–Dawley rats (weighing approximately 300–350 g) were purchased from Japan SLC Co., Ltd. (Hamamatsu, Japan). Each rat was anesthetized using sevoflurane and an intrasubcutaneous injection of three types of anesthesia (0.15 mg/kg medetomidine, 2 mg/kg midazolam, and 2.5 mg/kg butorphanol). Rats were intubated using a 16-gage catheter and ventilated with a small animal respirator (SN480-7; Shinano, Tokyo, Japan) at 70 cycles/min and a tidal volume of 10 mL/kg. After right anterior thoracotomy, the rat was managed with a positive end-expiratory pressure of 2-cm H_2_O. Sublobar resection (wedge resection) was performed using electrocautery coagulation at a power of 40 W, which was the minimum output for preventing air leakage after surgery, or a surgical stapler (ECHELON FLEX™ Powered Vascular Stapler; Ethicon, Cincinnati, OH, USA) at three points 2.5 cm from the peripheral summit of the right middle lobe (Fig. 1). Following carefully confirming no air leakage by the naked eye, the chest wall was closed without reinforcing the resected lung surface with any sheets or fibrin glue and placing a chest drain. After the chest wall and skin closure, all rats recovered quickly and were promptly extubated following subcutaneous injection of 5 mg/kg atipamezole and 5 mg/kg meloxicam. After surgery, the rats were maintained in an animal house with a 12-h light–dark cycle and allowed free access to water and food. All experiments included five rats in each group, except for the week 24 stapler model (N = 3). This study was approved by the Animal Care Committee of Shinshu University (no. 019111).

### Histopathological evaluation

The rats were euthanized using an overdose of sevoflurane at 2, 4, 8, 12, and 24 weeks. The lungs were perfused with phosphate-buffered saline through the main pulmonary artery after ligating the inferior vena cava and cutting both atria; thereafter, en-bloc removal of the lungs and heart was performed. Rat organs were fixed in a solution of 4% paraformaldehyde and an optimal cutting temperature compound at a 4:1 ratio, infused via the trachea at 20-cm H_2_O as previously described [14], and maintained overnight.

The coagulation area in the preserved lung lobe of the electrocautery model was evaluated using Heron’s formula [15]. The fixed lungs of both models were embedded in paraffin. During the procedures, the staplers of the stapler model were cut just below the staplers and removed from lung tissue after paraffin infiltration. Specimens embedded in paraffin were cut into 6-µm-thick sections for the evaluation of morphologic changes in the resected area of the electrocautery models. Histological evaluation was performed using hematoxylin and eosin (H&E), Hector Battifora mesothelial epitope-1 (HBME-1), and Elastica van Gieson (EVG) staining. HBME-1 and EVG staining were used to identify mesothelial cells and elastic fibers, respectively.

Immunofluorescent staining was performed to evaluate the alveolar epithelial cells (alveolar type I [AT1] and II [AT2] cells) to compare the electrocautery and stapler groups using 4-µm-thick sections. After deparaffinization, antigen retrieval (pH 9), and blocking of nonspecific binding with 5% normal donkey serum, sections were stained overnight at 4 ℃ with the following primary antibodies: HOPX (AT1 cell) mouse monoclonal antibody (1:100; sc-398703; Santa Cruz Biotechnology, Santa Cruz, CA, USA) and SFTPC (AT2 cell) rabbit polyclonal antibody (1:200; AB3786; Merck Millipore, Burlington, MA, USA). Alexa Fluor 488 donkey anti-rabbit IgG (1:500; A-21206; Thermo Fisher Scientific, Swedesboro, NJ, USA) and Alexa Fluor 555 donkey anti-mouse IgG (1:500; A-34570; Thermo Fisher Scientific) were incubated for 1 h.

**Fig. 2:**
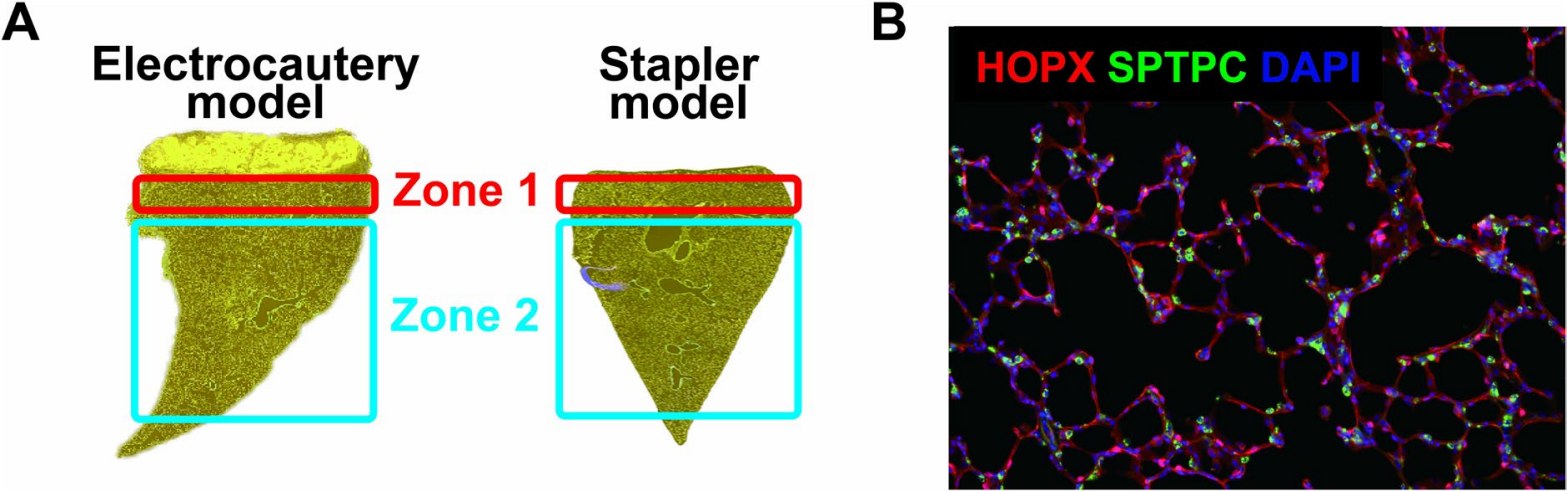
Definitions of the zones used for evaluation and a representative image of immunofluorescent staining. Zone 1 is the region directly below the resected area. Zone 2 is an area distinct from Zone 1 (**a**). HOPX-positive and SFTPC-positive cells indicating alveolar type I and type II cells, respectively (**b**)

The stained slides were scanned using the Vectra 3.0 spectral imaging system (PerkinElmer), and imaging and analysis were performed using InForm software. We captured 10 random images from each zone using the following definitions: Zone 1, below the resected area; Zone 2, separate from Zone 1; and Zone 3, the left lung, which was used as a control (Fig. 2a). These images were then used to measure the mean linear intercept (MLI) value by S.M., which was used to quantify lung expansion in each zone, as previously described (Supplementary Fig. S1) [16, 17]. A smaller MLI reflects less lung expansion, whereas a greater MLI indicates more lung expansion. In addition, we counted and compared the numbers of HOPX- and SFTPC-positive cells in each zone (Fig. 2b).

### Statistical analysis

All statistical analyses were performed using IBM SPSS Statistics version 27 software (IBM, Armonk, NY, USA). Paired comparisons between more than two groups were assessed using an analysis of variance, followed by Tukey’s post hoc test. For unpaired comparisons between more than two groups, Mauchly’s test was used. All reported values are presented as means, and the error bars in the figures represent the standard error of the mean. Statistical significance was defined as p < 0.05.

## Results

### Macroscopic and microscopic changes in the area resected using electrocautery

**Fig. 3:**
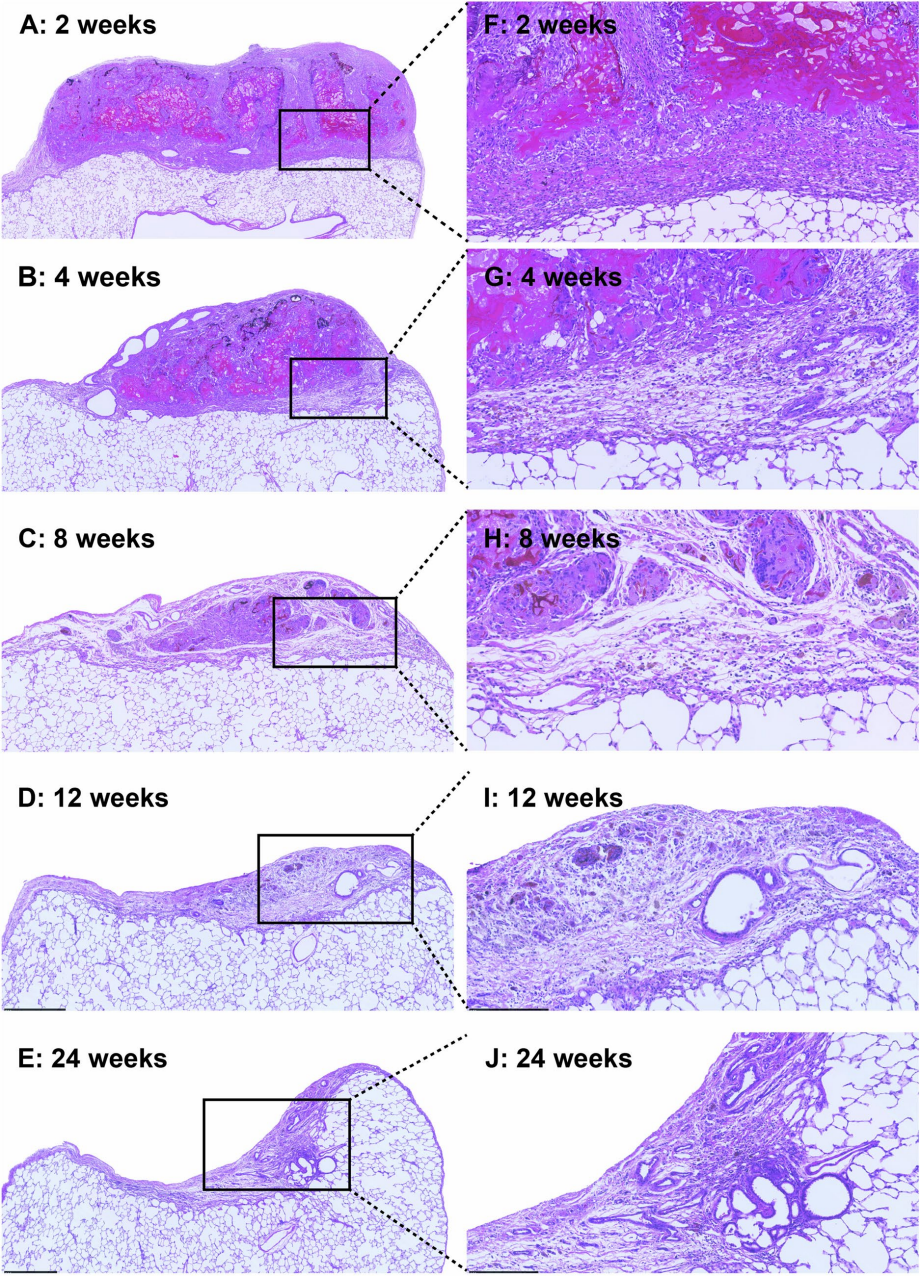
Hematoxylin and eosin staining of the lung area resected using electrocautery at 2, 4, 8, 12, and 24 weeks postoperatively under low-power magnification (**a**–**e**) and high-power magnification (**f**–**j**). Inflammatory cells infiltrated the area at week 2 (f). The population began to decrease and resolve from weeks 4 to 8 (**g** and **h**), followed by an increase in the number of vessels and bronchioles (**i** and **j**)

**Fig. 4:**
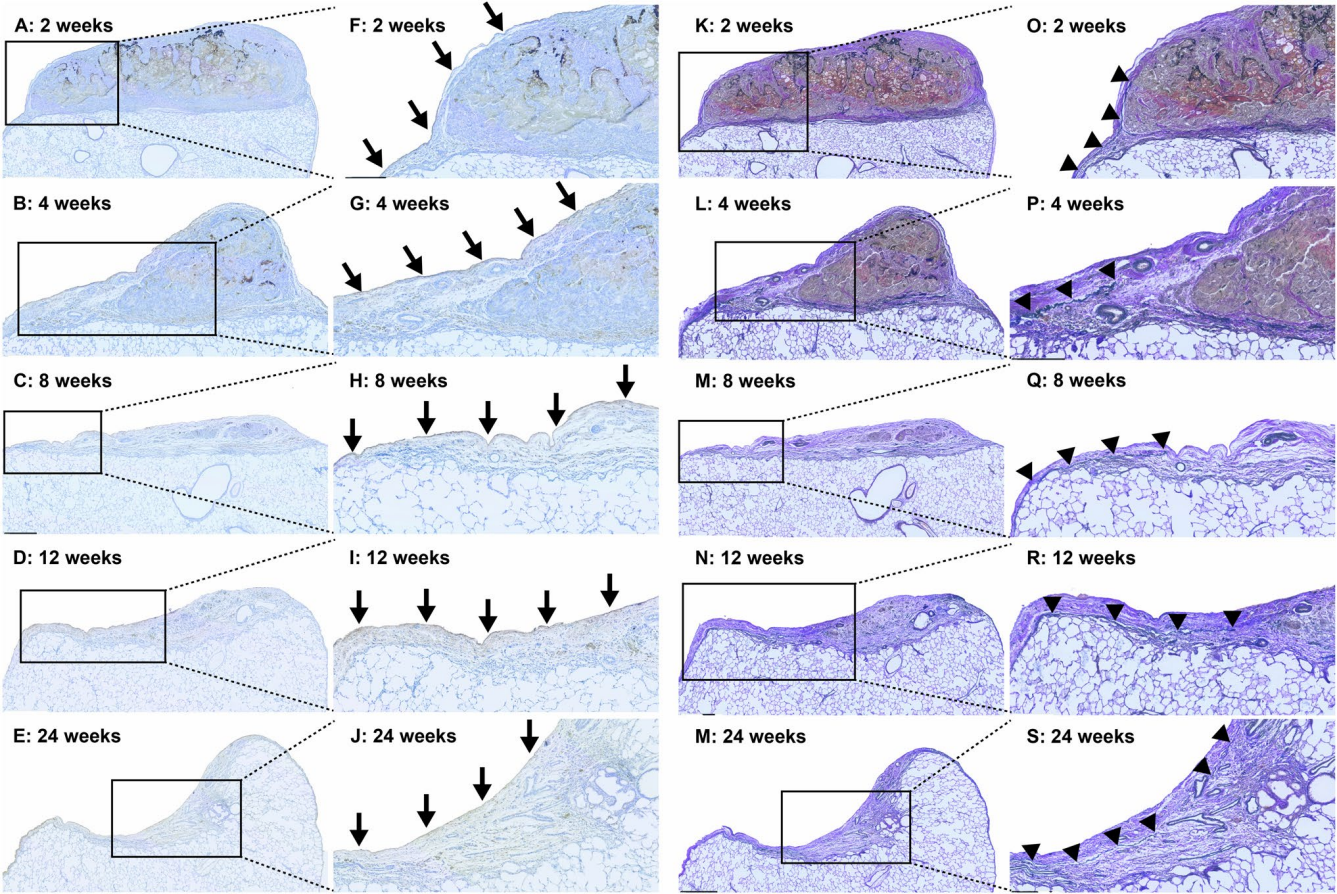
Hector Battifora mesothelial epitope-1 (**a**–**j**) and Elastica van Gieson staining (**k**–**s**) of the lung area resected using electrocautery at postoperative weeks 2, 4, 8, 12, and 24 under low-power magnification (**a**–**e** and **k**–**m**) and high-power magnification (**f**–**j** and **o**–**s**). Mesothelium cells (arrow) covered the entire resected area at week 2 (f). This repair persisted from weeks 4 to 24 (**g**–**j**). Elastic fibers (arrowhead) began extending from both sides between weeks 2 and 24 (**o**–**s**)

Supplementary Fig. S2a shows macroscopic changes in the coagulated area resected using electrocautery. The mean coagulated area, which was calculated using Heron’s formula, significantly decreased from week 2 to week 24 (Supplementary Fig. S2b). These changes were assessed by H&E staining, and the time-dependent histologic findings are summarized in Fig. 3. At week 2, neutrophilic cells, macrophages, and fibroblasts infiltrated the resected area (Fig. 3f). These cells began to decrease from weeks 4 to 8 (Fig. 4g and h), and the number of vessels and bronchioles in the area increased from weeks 12 to 24 (Fig. 3i and j). To evaluate the pleural region, the repair processes were assessed using mesothelial cells and elastic fibers, which constitute the outer- and innermost layers of the pleura, respectively (Fig. 4). Mesothelial cells completely covered the entire outermost layer of the resected area, which were continuous from the visceral pleura without adhesion to any chest-wall tissues, 2 weeks after surgery (Fig. 4f), and this coverage persisted from weeks 4 to 24 (Fig. 4g–j). By contrast, the elastic fibers gradually extended from both sides over time (Fig. 4o–r), finally growing to the point of connection from both sides at week 24 (Fig. 4s).

### Comparison of the MLI and alveolar epithelial cells in the preserved lobe of the electrocautery and stapler groups

**Fig. 5:**
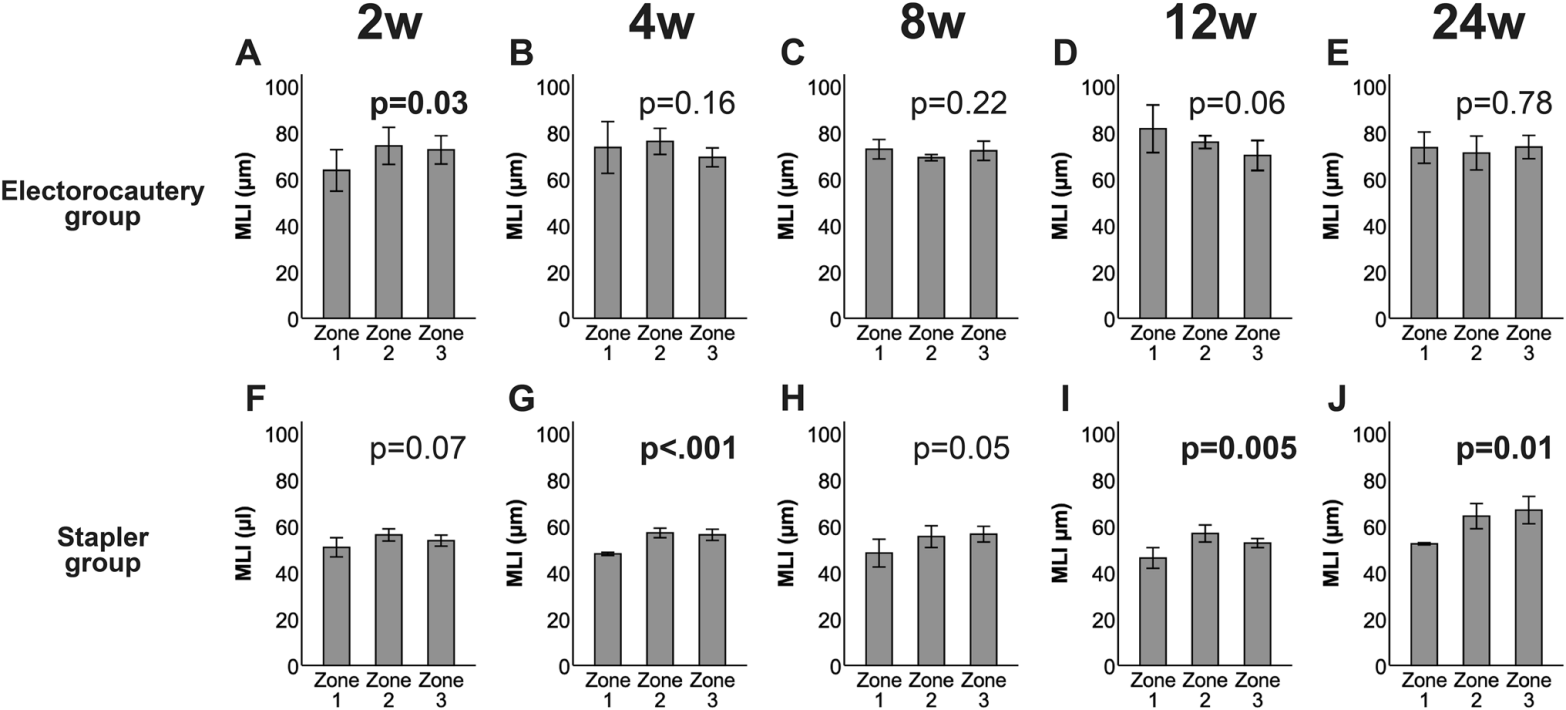
Postoperative alveolar size after sublobar resection in the electrocautery (**a**–**e**) and stapler groups (**f**–**j**), as assessed using the mean linear intercept (MLI). The MLI in Zone 1 of the electrocautery group was significantly smaller than those in the other zones at week 2 (**a**), and there was no difference in the MLIs of all zones from weeks 4 to 24 (**b**–**e**). The MLI in Zone 1 of the stapler group remained smaller across the different timepoints (**f**–**j**). Data are presented as the mean, and the bars indicate the standard deviation

In the electrocautery group, the MLI was smaller in Zone 1 than in the other zones at week 2 (Fig. 5a), but there was no difference in the MLIs of all zones from weeks 4 to 24 (Fig. 5b–e). By contrast, Zone 1 in the stapler group consistently had a smaller MLI than those in the other zones from weeks 2 to 24 (Fig. 5f–j). Supplemental Fig. S3 demonstrates the results of the MLI from weeks 2 to 24 in each zone according to the two groups.

**Fig. 6:**
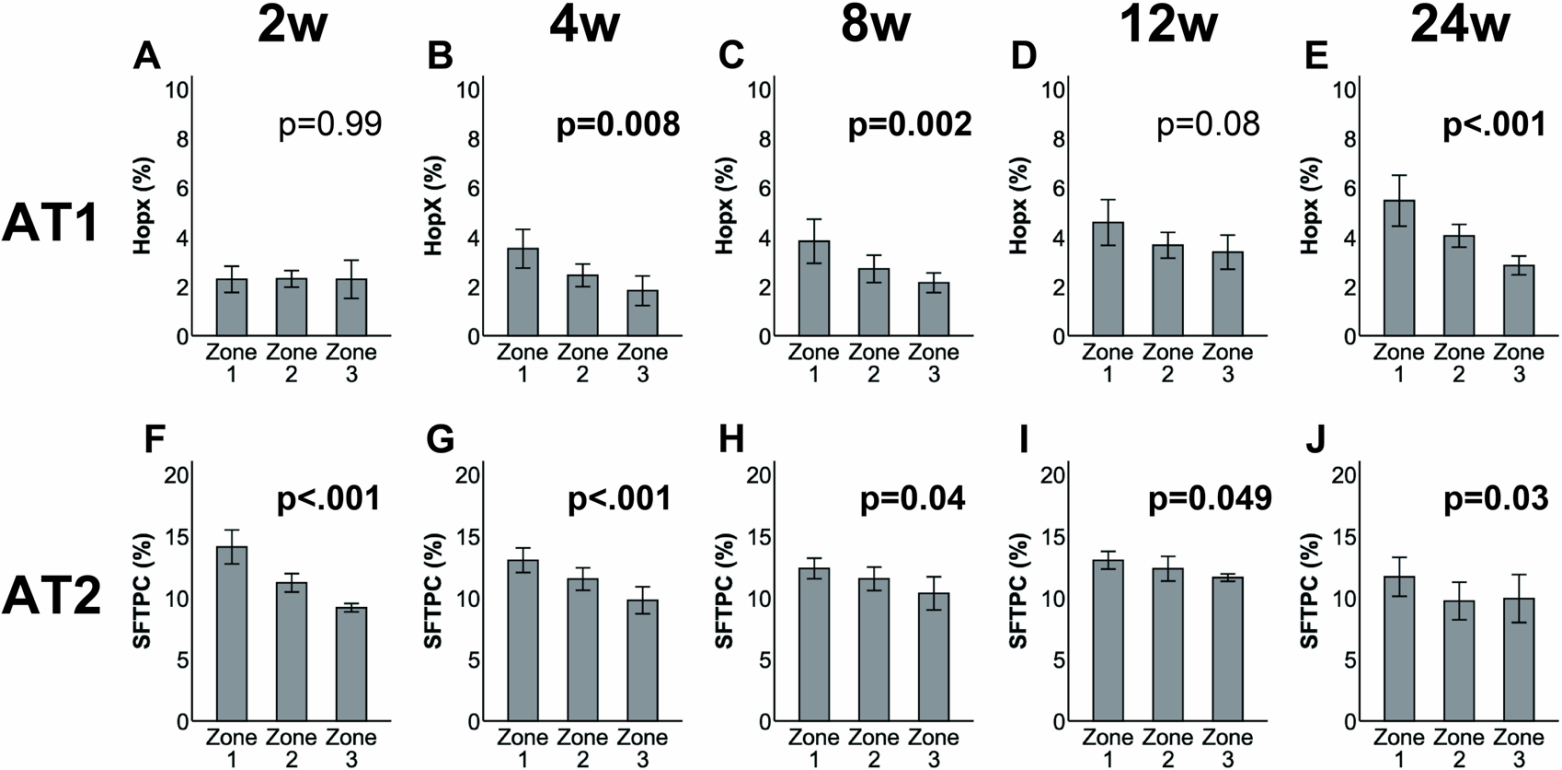
Quantification of alveolar type I (AT1) and alveolar type II (AT2) cells after sublobar resection using electrocautery. The number of AT1 cells in Zone 1 was higher than those in the other zones from weeks 4 to 24 (**b**–**e**). The number of AT2 cells showed an increase over time (**f**–**j**). Data are presented as the mean, and bars indicate the standard deviation

**Fig. 7:**
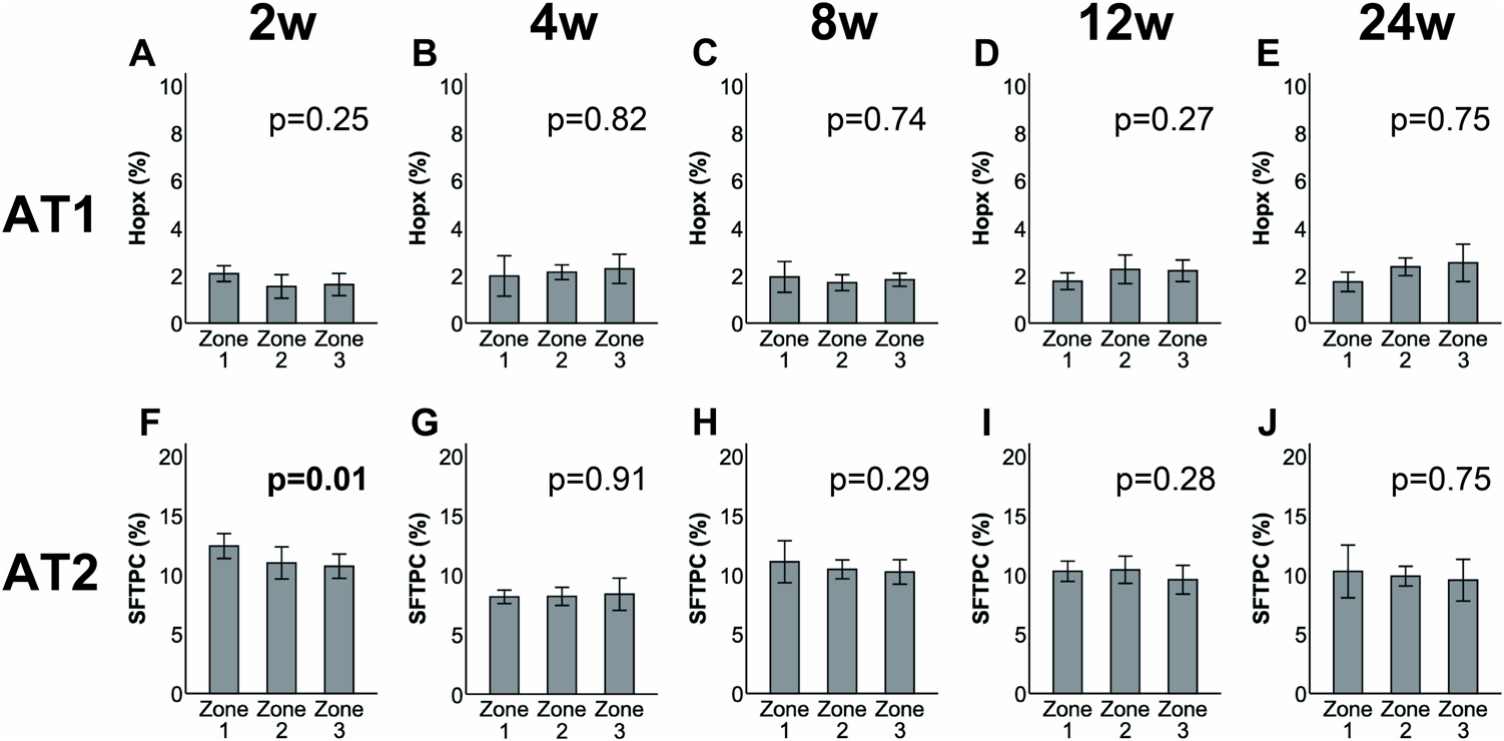
Quantification of alveolar type I (AT1) and alveolar type II (AT2) cells after sublobar resection using a stapler. An increase in the number of AT2 cells in Zone 1 was only observed in week 2 (f). No significant changes in the numbers of AT1 (**a**–**e**) and AT2 cells occurred thereafter (**g**–**j**). Data are presented as the mean, and bars indicate the standard deviation

Regarding alveolar epithelial cell proliferation, the electrocautery group exhibited an increase in AT2 cells in Zone 1 compared to the other zones at week 2 after surgery (Fig. 6f). This increase continued until week 24 (Fig. 6g–j), followed by an increase in AT1 cells in Zone 1 from weeks 8 to 24 (Fig. 6b–e). By contrast, the stapler group only exhibited an increase in AT2 cells in Zone 1 at week 2 (Fig. 7f), with no significant changes in AT1 (Fig. 7a–e) or AT2 cells thereafter (Fig. 7g–j). Supplemental Fig. S4 and S5 demonstrates the results of the AT1 and AT2 cells, respectively, from weeks 2 to 24 in each zone according to the two groups.

## Discussion

During this study, we analyzed the histologic changes in a rat sublobar resection model, specifically focusing on the electrocautery method. In addition, we conducted a comparative examination of the histologic differences in the preserved lobes of the electrocautery and stapler groups. Although previous studies investigated the preserved lung volume after sublobar resection using 3D-CT imaging [18] and excised pig lungs [19], to our knowledge, this is the first study evaluating the histologic characteristics of the preserved lungs after sublobar resection and comparing the findings between two types of sublobar resection models over long-term (6-month). The novel findings associated with the electrocautery model compared with the stapler model were as follows: a pulmonary self-repair phenomenon was observed in the resected area; lung expansion after surgery initially exhibited a brief period of collapse, followed by recovery; and the population of alveolar epithelial cells increased in the preserved lung lobes. These results may indicate a potential clinical advantage of promoting postoperative lung conditions and functions when dividing the lung parenchyma and intersegmental plane using electrocautery during wedge resection and segmentectomy.

The lungs can be repaired and regenerated because of their continuous defense against environmental threats, including chemicals and infectious agents, leading to significant lung damage. Previous studies of animal models of diseases, such as acute lung injury and fibrotic lung disease [20–22], have examined the mechanisms involved in the repair and regeneration processes after various types of injuries. By contrast, compensatory lung growth, rather than repair or regeneration, has been observed in adult humans after lung-resection surgery [14, 23, 24]. It is considered that this phenomenon involves the enlargement of the remaining lung tissue within the chest cavity, effectively filling the space created by surgical removal of the lung. However, few studies have examined the morphologic changes in the preserved lung lobe after sublobar resection. During this study, we investigated the macroscopic and microscopic findings in the area resected using electrocautery. Notably, the macroscopic coagulated area decreased significantly from 2 to 24 weeks postoperatively, indicating the occurrence of a restorative phenomenon in the resected area. H&E staining revealed that during the early postoperative period, the resected area exhibited infiltrating inflammatory cells, which appeared to correspond to the inflammation and proliferation stages of wound healing. Subsequently, absorption of cellular components and growth of vessels and bronchioles occurred, indicating the maturation stage of the wound healing phase; this was the first examination to show the self-repair processes in the resected lung after surgery using electrocautery.

Little is known regarding the repair process of the pleura after lung resection using electrocautery. This study examined two distinct repair processes involving mesothelial cells and elastic fibers, which comprise the outermost and inner layers of the pleura, respectively. We found that the mesothelial cells completely covered the outermost layer of the resected area by week 2 after surgery. By contrast, the elastic fibers were only gradually repaired at both edges by week 24. Although these findings are similar to those of a previous study that examined the healing process of visceral pleural defects covered by TachoSil [25], our study is the first to report that mesothelial cells and elastic fibers of the pleura exhibit rapid and slow repair capacities, respectively, in lung areas resected using electrocautery.

Furthermore, using the sublobar resection model, we compared the postoperative MLI, which is an indicator of lung expansion, and alveolar epithelial cell proliferation in the preserved lung lobe of the two groups. Our study revealed that MLI in the resected area of the stapler group remained small. By contrast, the electrocautery group had only a small MLI in the resected area during the early postoperative period; thereafter, the MLI became and remained larger, similar to that in other regions. In a prior study, Asakura et al. used a porcine model to demonstrate that staplers reduced preserved lung expansion compared with the use of scissors during segmentectomy [19]. These findings suggest that electrocautery may be more beneficial than stapler use for postoperative lung expansion. In addition, we observed an increase in the AT2 cells below the resected area in the electrocautery group from 2 weeks after surgery, followed by an increase in the AT1 cells until 24 weeks after surgery. Previous studies have reported that AT2 cells can function both as a self-renewing stem cell-like population and regenerating AT1 cells after injury [26–28], which may be linked to our findings of alveolar cell proliferation in the electrocautery group. Although excessive inflammation is associated with irreversible lung diseases such as emphysema and lung fibrosis, appropriate inflammation is essential for regeneration as it removes harmful pathogens, as well as debris derived from apoptotic and necrotic cells, leading to repair and regeneration of the lung epithelium [29, 30]. Therefore, lung resection performed using electrocautery, which triggers inflammation, may offer advantages in the repair and regeneration processes, leading to different long-term morphologic changes in the preserved lung after surgery compared to the stapler method. Furthermore, the continuous increase in AT2 cells, which was only observed in the electrocautery group, may lead to early-phase recovery of the MLI in the resected area by secreting the surfactant apoprotein.

Despite the strengths of this study, a few limitations should be mentioned. First, our study lacked a direct comparison of the alveolar size and the rate of alveolar epithelial cells between the electrocautery and the stapler groups. However, conducting such an analysis would only provide results on the superiority between the two groups at each point, making it challenging to evaluate these results over time. Furthermore, including a sham group, where only thoracotomy is performed, would be necessary for a more comprehensive analysis. Therefore, in this study, we focused on the changes in these findings over time, using the individual left lung as a reference, and demonstrated differences and changes over time in lung expansion and alveolar epithelial cells in the preserved lobe following sublobar resection between these two groups. Second, experiments were conducted using a rat model of normal adult lungs. Thus, we could not investigate the postoperative processes of lung resection under abnormal lung conditions such as lung emphysema and interstitial pneumonia. Third, our sublobar resection model did not divide the intersegmental plane, and the amount of lung tissue was relatively small (approximately one-third of the rat middle lobe). However, our model shares similarities with segmentectomy in terms of evaluating the preserved lung after surgery, and this model was the most reproducible in terms of procedural safety and survival rates among several attempts of sublobar resections in other lobes. Fourth, we could not evaluate the histologic findings in the peri-staple area in the stapler model because the paraffin block, including the staplers, cannot be cut using the microtome knife in the process of tissue preparations; thus, the specimen was cut just below the staples before the paraffin embedding to make thin sectioning. Fifth, we analyzed the stapler model at 24 weeks with a minimum number of rats (n=3) because inappropriate specimens were unexpectedly obtained during the tissue preparation. Finally, the functional and physiologic impact after segmentectomy, including both the electrocautery and staple methods, need to be verified through assessments such as physical activity, changes in the skeletal muscle, and cytokine levels in the blood. These limitations should be considered when interpreting the results. Further research is required to address these factors comprehensively.

## Conclusion

Our sublobar resection model using electrocautery demonstrated potential for lung self-repair after surgery, greater lung expansion, and an increased number of alveolar cells in the preserved lung lobe compared to the stapler method. These findings may indicate that the electrocautery lung-dividing method provides advantages in terms of the postoperative repair and regeneration processes, ultimately contributing to improved lung conditions and functions.

## Supplementary Information

Below is the link to the electronic supplementary material.

Supplementary file1 (TIF 19991 KB)

Supplementary file2 (TIF 4550 KB)

Supplementary file3 (TIF 6575 KB)

Supplementary file4 (TIF 5532 KB)

Supplementary file5 (TIF 6225 KB)

Supplementary file6 (DOCX 17 KB) 

## Data Availability

The data will only be shared on reasonable request to the corresponding author.
