# Supplementary figures and images for: Long-term pulmonary repair in rat lungs after sublobar resection: electrocautery versus stapler methods
Source: Gen Thorac Cardiovasc Surg. 2024 Oct 28;73(7):497–505. doi: 10.1007/s11748-024-02098-8 (PMC12174207; doi:10.1007/s11748-024-02098-8)

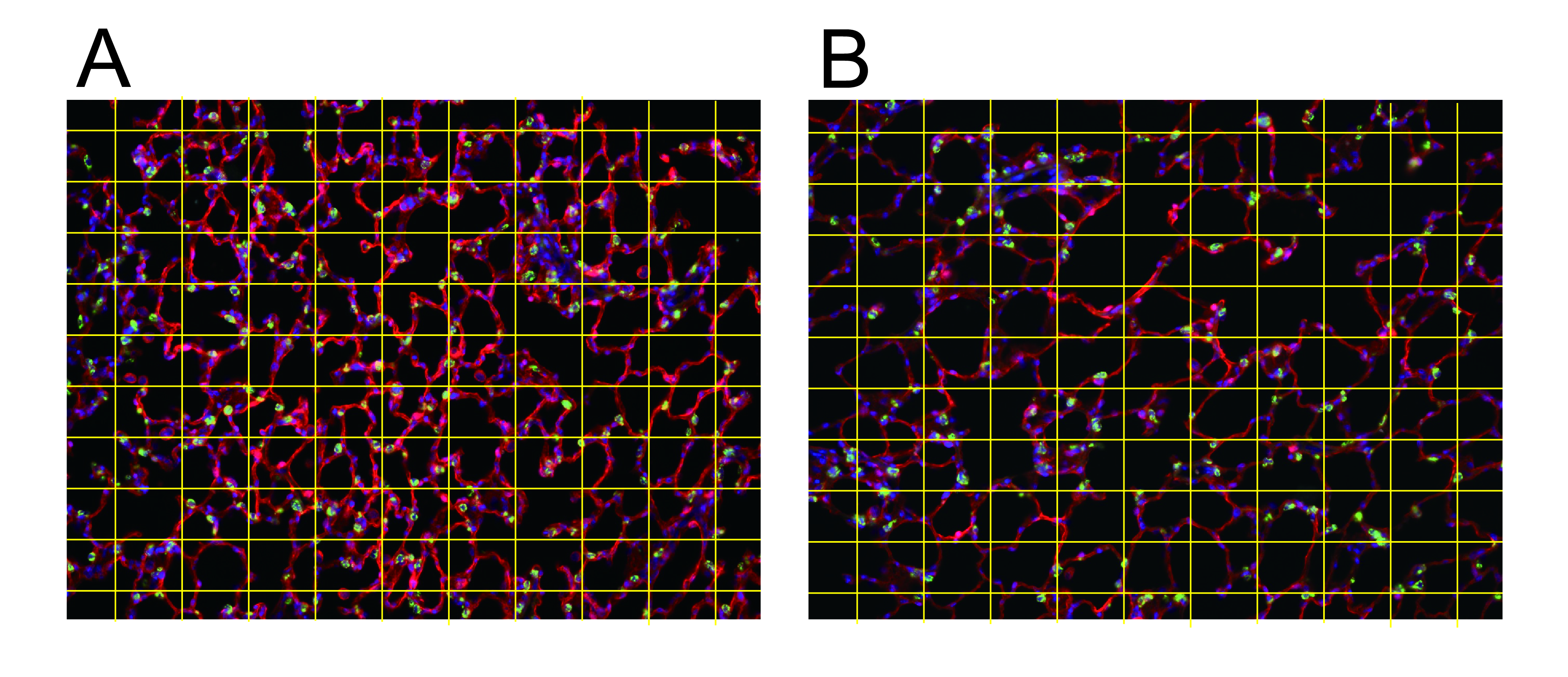

Supplement: Supplementary file 1 — Supplementary file1 (TIF 19991 KB) [file 11748_2024_2098_MOESM1_ESM.tif]

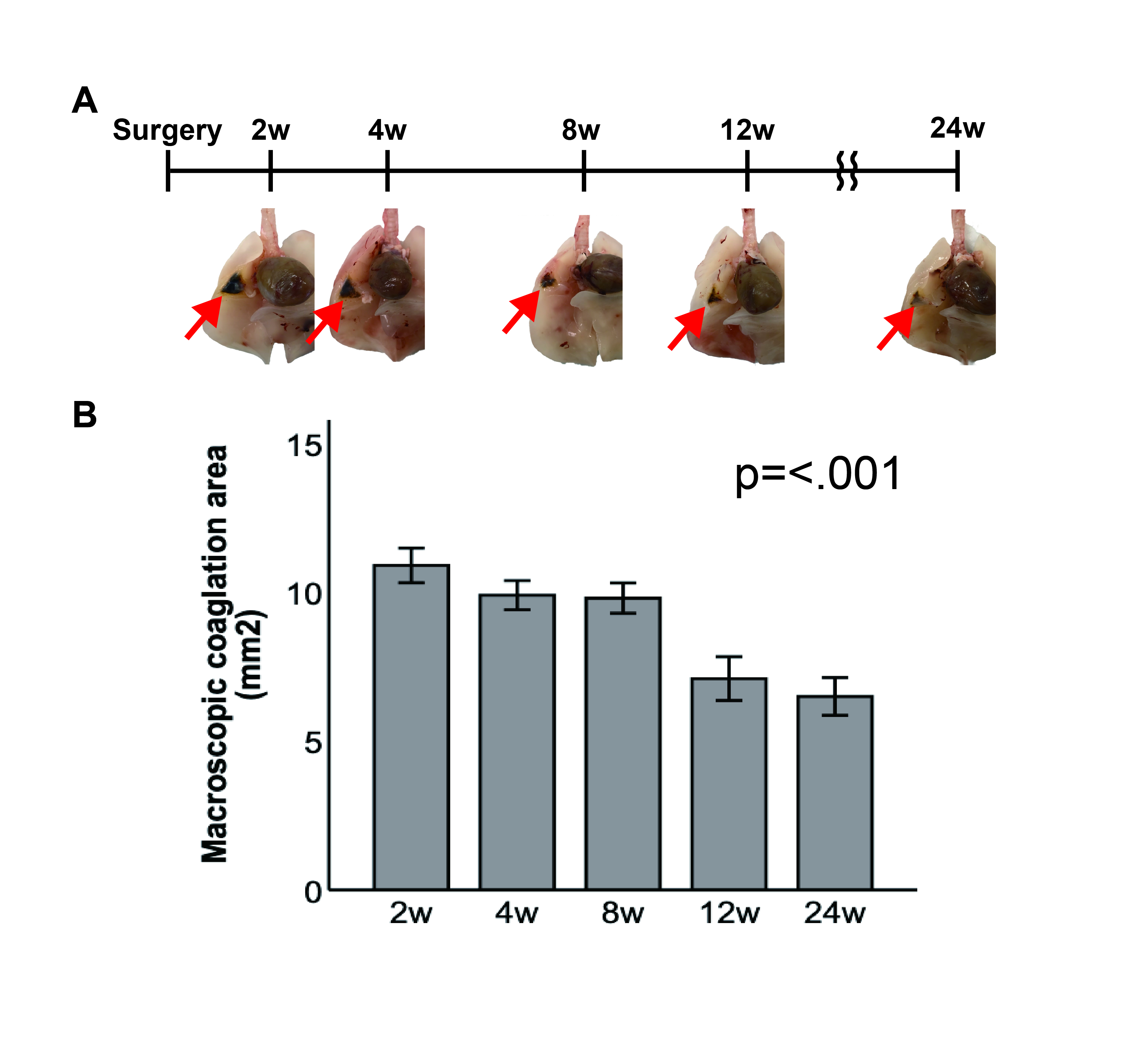

Supplement: Supplementary file 2 — Supplementary file2 (TIF 4550 KB) [file 11748_2024_2098_MOESM2_ESM.tif]
