# Supplementary material for: Long-term pulmonary repair in rat lungs after sublobar resection: electrocautery versus stapler methods
Source: Gen Thorac Cardiovasc Surg. 2024 Oct 28;73(7):497–505. doi: 10.1007/s11748-024-02098-8 (PMC12174207; doi:10.1007/s11748-024-02098-8)
Supplement: Supplementary file 6 — Supplementary file6 (DOCX 17 KB) [file 11748_2024_2098_MOESM6_ESM.docx]

**Supplemental Fig. S1** Comparison of smaller (a) and larger (b) mean linear intercepts (MLIs) of representative cases. A transparent sheet with 10 horizontal and vertical lines was laid over the images. The intercepts of the alveolar walls with these lines were counted

**Supplemental Fig. S2** Postoperative macroscopic changes in the area resected using electrocautery. Arrows indicate the coagulated area (a), which decreased significantly over time (b)

**Supplemental Fig. S3** Postoperative alveolar size after sublobar resection in Zone 1 to 3 of the electrocautery (a, c, e) and stapler groups (b, d, f), as assessed using the mean linear intercept (MLI)

**Supplemental Fig. S4** Quantification of alveolar type I (Hopx) in Zone 1 to 3 after sublobar resection using the electrocautery (a, c, e) and stapler methods (b, d, f). Data are presented as the mean, and bars indicate the standard deviation

**Supplemental Fig. S5** Quantification of alveolar type II (SFTPC) in Zone 1 to 3 after sublobar resection using the electrocautery (a, c, e) and stapler methods (b, d, f). Data are presented as the mean, and bars indicate the standard deviation
